# Thermal and Kinetic Studies on Biomass Degradation *via* Thermogravimetric Analysis: A Combination of Model-Fitting and Model-Free Approach

**DOI:** 10.1021/acsomega.1c02937

**Published:** 2021-08-16

**Authors:** Tolu Emiola-Sadiq, Lifeng Zhang, Ajay K. Dalai

**Affiliations:** Department of Chemical and Biological Engineering, University of Saskatchewan, Saskatoon S7N 5A8, Canada

## Abstract

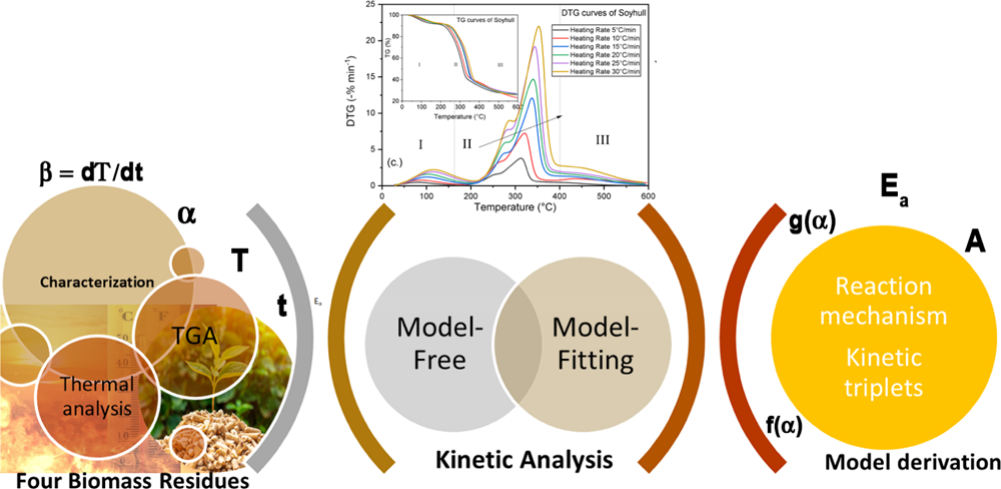

Thermal degradation behavior and kinetics of two agricultural (soy and oat hulls) and two forestry biomass (willow and spruce) residues were investigated using a unique combination of model-fitting and model-free methods. Experiments were carried out in an inert atmosphere at different heating rates. Both single step and multistep models were explored in deriving activation energies, frequency factors, and mechanisms of all four biomass residues. For the single step models, activation energy values ranged from 107.2 kJ/mol for willow and 139.7 kJ/mol for soy hull, and the frequency factors for both materials were 1.1 × 10^9^ and 2.66 × 10^12^ s^–1^, respectively. The multistep models gave further insight into the different mechanisms across the full degradation spectrum. There was an observed difference between the number of distinct steps/mechanisms for the agriculture-based versus wood-based biomass materials, with pyrolysis occurring in three distinct steps for the agricultural biomass residues while the woody residues degraded in two steps. The difference in the number of distinct steps can be attributed to the composition and distribution of components of the biomass, which would differ based on the nature and source of the biomass.

## 1 Introduction

The utilization of renewable materials for energy generation has garnered much attention in the past decade due to the negative environmental impact of nonrenewable energy sources and the inevitable decline in accessible fossil fuel resources. Biomass remains a potential source of alternative energy as we move toward a more sustainable and cleaner energy future. The ubiquitous nature of biomass—with sources ranging from lignocellulosic materials like forestry and agricultural residues, to energy crops, municipal wastes, industrial and animal residues, coupled with having a net carbon-neutral effect on the environment—makes it a viable option for use as a renewable energy source. Lignocellulosic biomass is used as feedstock for various thermochemical conversion processes: pyrolysis for bio-oils and char production,^1^ gasification for gaseous fuels and other products,^2^ and combustion, with the latter being arguably the most popular carbon-based conversion process. Lignocellulosic biomass can be further classified as either being derived from agricultural sources or wood-based sources. The agricultural biomass residues are essentially byproducts from different crops and grasses while woody biomasses are mainly from forest or woody materials. Thus, waste from either source could serve as feedstock for the thermochemical conversion processes mentioned earlier. However, because biomass comes in various forms and shapes, one major concern is the slightly complex heterogeneous composition, which makes for multiple reactions occurring during the conversion process. The extent of progress of these multiple reactions or *vice versa* is dependent on several parameters and conditions such as reaction temperature, residence time, feed rate, and heating rate.^2^ These parameters are in turn dependent on the kinetic rates of the reaction. It is therefore important to study the thermal decomposition kinetics of biomass, as it is essential to predict the conversion process, understand the mechanisms involved, evaluate kinetic models, and optimize the design of the reactor systems required for its conversion.

Thermogravimetric analysis (TGA) is a powerful technique that measures mass changes of a material with temperature. At very low heat- and mass-transfer effects, this mass change gives detailed information about thermal stability, reactivity, reaction mechanism, decomposition kinetics, content determination, and compositional analysis.^3^ Due to its versatility and efficiency, TGA has been used extensively for kinetic studies involving devolatilization of different compounds and materials. This technique provides the thermal response and degradation of materials as a function of temperature over a period and controlled temperature program. It directly measures the mass of a sample, as it is heated, cooled, or held at a definite temperature in a defined atmosphere. Thus, TGA has been used to study materials like polycarbonates,^4^ microalgae,^5^ tar sands,^6^ coal,^7^ different biomass residues,^8−12^ and biomass/coal blends^13,14^ among others.

Nonisothermal or linear heating rate TGA methods involve heating the material to a desired temperature at a desired constant heating rate with time. This method has the advantage of being applicable to complex multistaged reactions, with better reliability and replicability compared to isothermal methods, and thus reactions can attain a full conversion. Though it has a slight disadvantage of being time-consuming by requiring multiple experiments at different heating rates, nonisothermal TGA has been shown to be very useful and one of the best methods for studying the thermal decomposition kinetics of biomass materials. Isoconversional models (differential or integral) allow for the estimation of the activation energy as a function of temperature at a certain conversion without previous assumptions on the reaction mechanism. They are therefore referred to as model-free methods as no prior model assumptions are required. Based on the existing literature, the most widely used differential method is the Friedmann method,^15^ which has the advantage of being accurate and not limited to heating rate variation. Integral methods include the Flynn–Wall–Ozawa (FWO),^16^ Kissinger–Akahira–Sunose (KAS),^17,18^ Starink,^19^ and Vyazkovin^20^ methods. Isoconversional methods are often carried out with at least three different heating rates, with the assumption that the reaction rate at a conversion value is a function of temperature. The resulting activation energy (*E*_α_) versus conversion (α) data make it possible to derive the pre-exponential factor (*A*) by using either master plots,^21^ fitting the reaction using different models (*f*(α) or *g*(α))^22^ or as initial values for iterative steps required for model fitting.^23^ Isoconversional models are simple and suitable for complex processes where prior knowledge of the reaction mechanism is unavailable due to the multiple reaction mechanisms inherent in those processes.^25^ Isoconversional models therefore eliminate the challenge associated with prior selection or assumption of a reaction model and are well suited for qualitative results rather than precise quantitative evaluation. As no assumption is made in determining the kinetic models, there is a better chance of differential methods being more accurate than integral isoconversional methods. Nevertheless, this presumed accuracy is greatly reduced in such scenario when the activation energy *E*_α_ is dependent on the heating rate, causing difficulties in the baseline experimental definition. Although model-free methods are quite robust for nonisothermal kinetics, they are mostly empirical and hardly give any physical or kinetic meanings during thermal analysis. As model-fitting methods require using a reaction mechanism or model, which gives further information on the kinetics of the thermal degradation process, a combination of both methods is therefore considered.

Some reaction kinetic models often used in solid-state reaction kinetics^10,11,24^ are summarized in Table 1.

**Table 1:** Theoretical Kinetic Models Showing Both Differential and Integral Forms

Model	differential form *f*(α) = 1/*k* (dα/d*t*)	integral form *g*(α) = *kt*
Nucleation Models		
power law (P2)	2α^1/2^	α^1/2^
power law (P3)	3α^2/3^	α^1/3^
power law (P4)	4α^3/4^	α^1/4^
Avrami–Erofeev (A2)	2(1 – α) [−ln(1 – α)]^1/2^	[−ln(1 – α)]^1/2^
Avrami–Erofeev (A3)	3(1 – α) [−ln(1 – α)]^2/3^	[−ln(1 – α)]^1/3^
Avrami–Erofeev (A4)	4(1 – α) [−ln(1 – α)]^3/4^	[−ln(1 – α)]^1/4^
Geometrical Contraction Models		
contracting area (R2)	2(1 – α)^1/2^	1 – (1 – α)^1/2^
contracting volume (R3)	3(1 – α)^2/3^	1 – (1 – α)^1/3^
Diffusion Models		
1D diffusion (D1)	1/(2α)	α^2^
2D diffusion (D2)	–[1/ln(1 – α)]	[(1 – α) ln(1 – α)] + α
3D diffusion––Jander (D3)	[3(1 – α)^2/3^]/[2(1 – (1 – α)^1/3^)]	[1 – (1 – α)^1/3^]^2^
Ginstling–Brounshtein (D4)	3/[2((1 – α)^1/3^ – 1)]	1 – (2/3)α – (1 – α)^2/3^
Reaction Order Models		
zero order (F0)	1	α
first order (F1)	(1 – α)	–ln(1 – α)
second order (F2)	(1 – α)^2^	[1/(1 – α)] – 1
third order (F3)	(1 – α)^3^	1/2[(1 – α)^−2^ – 1]

The kinetic parameters are often derived by fitting the experimental data of the conversion dependence of the reaction rate to the assumed model. This model-fitting is usually done by reducing the difference between the experimentally measured and calculated data on the reaction rate using linear or nonlinear regression analysis. Lignocellulosic biomass is mainly composed of a complex mix of three pseudocomponents: hemicellulose, cellulose, and lignin. Multistep reaction models are used to simulate biomass decomposition kinetics based on these three pseudocomponents. The mechanism of such multistep models could be parallel, interactive, or sequential.^25^ Another method called the distributed activated energy model (DAEM)^26^ is such that the activation energy is presumed to follow a continuous distribution as the number of first-order parallel reactions tend to infinity.^27^

Various researchers have studied different biomass materials using different methods, and the results are shown in Table 2.

**Table 2:** Summary of the Literature Review of TGA Studies on Different Feedstocks, Methods Used, and Results

		biomass type and results			
TGA studies	method	material	*E*_a_ (kJ/mol)	*A* (1/s)	reaction mechanism/order
Damartzis *et al.*(8)	nonisothermal@5–30 °C/min,isoconversional (FWO, KAS) methods	cardoon stems	29.0–229.70	1.8 × 10^1^ to 2.3 × 10^19^	*n*th order = 8.39–9.21
		cardoon leaves	36.0–350.07	2.8 × 10^1^ to 4.23 × 10^31^	*n*th order = 13.65–14.85
Gogoi *et al.*(9)	nonisothermal@5–20 °C/min, isoconversional methods	messua ferrea	180–380	1.74 × 10^18^ to 5.78 × 10^23^	nucleation and 3D diffusion mechanism
Hu *et al.*(10)	nonisothermal@5–20 °C/min	pinewood	152.43–210.39	3.22 × 10^12^ to 1.87 × 10^13^	diffusion, nucleation
	model-free (MF) and DAEM	bamboo	167.08–203.57	1.81 × 10^11^ to 1.51 × 10^14^	random scission, second and third order
Rueda-Ordóñez *et al.*(28)	nonisothermal@1.25–10 °C/min, isoconversional	sugarcane straw	154.1–177.8	1.82 × 10^9^	2D dimensional diffusion
Huang *et al.*(29)	nonisothermal@5–30 °C/min, KAS, FWO, Coats–Redfern	soybean straw	154.15–156.22	4.26 × 10^13^ to 1.09 × 10^16^	*n*th order = 8.19–17.31
Mishra *et al.*(30)	model-free, nonisothermal 5–40 °C/min	palm kernel shell	88–146	3.00 × 10^7^ to 6.00 × 10^12^	3.3–6.9 order
Lopes *et al.*(31)	nonisothermal@5–15 °C/min, isoconversional method	guarana seed residue	52–171	6.55–9.40 × 10^4^	parallel reactions, first order and second order
Collazzo *et al.*(32)	nonisothermal@5–50 °C/min, isoconversional methods KAS, FWO	elephant grass	46.5–185.28	0.6 × 10^1^ to 2.7 × 10^9^	diffusion and order-based mechanisms
Kaur *et al.*(33)	nonisothermal@5–40 °C/min, isoconversional KAS, FWO methods	castor residue	165.85–167.10	7.68 × 10^7^ to 7.92 × 10^18^	no mechanism or order stated
Walkowiak and Bartkowiak^34^	isothermal@270–330 °C, He atmosphere	raw and torrefied willow	138.1–227.3	1.19 × 10^10^ to 1.27 × 10^19^	first order, diffusion D3
Ondro *et al.*(35)	nonisothermal@5–30 °C/min, isoconversional Friedman, KAS, FWO methods	spruce wood	168.6–196.5		

Literature survey, some of which are presented in Table 2, showed that the Starink and advanced Vyazkovin isoconversional methods are less often used in investigating kinetic triplets compared to the Friedmann, FWO, and KAS models.

Further survey on the existing literature on pyrolysis and kinetics of certain biomass types was also conducted. Toro-Trochez *et al.*(36) conducted TGA characterization and pyrolysis of soy hulls to obtain the physicochemical properties of both the raw hulls and the final products, as well as the yields of the pyrolytic products at three different temperatures. Santana *et al.*(37) investigated the effect of impregnating metal chlorides on thermal degradation and the pyrolytic products obtained from soy hull pyrolysis. By varying the pyrolysis temperature between 400 and 500 °C, Santos *et al.*(38) studied the effect of the different temperatures on the properties of the pyrolytic products of oat hulls. González *et al.*(39) studied the effects of heating rate, temperature, residence time, and nitrogen flux on the physicochemical properties of oat hull-derived biochar.

It was observed that the two agricultural biomass materials, soy hulls and oat hulls, have not been thoroughly investigated, especially in terms of both their thermal degradation and kinetic parameters compared to other biomass materials.

Therefore, this work used a unique combination of both model-free and model-fitting techniques to explore and compare the thermal decomposition pattern and kinetic parameters of two agricultural and two wood-based biomass residues. Although the previous section mentioned the accuracy of differential models, it should be noted that integral models cannot be considered to be less precise than these differential methods, and so kinetic modeling with both methods is used in our study.^24^ A comparison between the results from different models used for the agriculture- and wood-based biomass materials was also conducted. The results would prove useful in further understanding, applying, and predicting the kinetics and thermal decomposition behavior for biomass residues from either similar or different feedstocks.

## 2 Experimental Section

### 2.1 Materials

Four different lignocellulosic biomass materials, two agriculture residues (soy hull and oat hull) and two forestry biomass residues (willow sawdust and spruce wood), were used in this study. The soy hull samples were supplied by Otter Farms, Aldergrove, BC, oat hull from Richardson Milling, spruce from Vanderwell Ltd., Alberta, and the willow samples from Warman Truss, Saskatchewan, Canada. The samples were prepared based on ASTM standards E1757-19 for compositional analysis. All biomass samples were of uniform size of about 15 ± 1.2 mg to reduce the effect of nonuniformity on the experiment. Proximate, ultimate, and compositional analyses were carried out to give further insights into the type of materials used in this work.

### 2.2 Methods

For proximate analysis, moisture content was derived according to ASTM E871-82 (2019) standards. The volatile matter and ash contents were derived according to ASTM E872-82 (2019) and ASTM E1755-01 (2015), respectively. The fixed carbon was estimated by mass difference. Ultimate analysis was carried out to derive the carbon, hydrogen, nitrogen, and sulfur contents for each of the four biomass materials. These were measured by using a PerkinElmer CHNSO analyzer (Vario El III, Elementar Americas Inc. NJ). Compositional analysis was carried out with an Ankom A2000 analyzer using acid and neutral detergent fiber methods. The higher heating values (HHV) were derived *via* bomb calorimetry (Parr 6400, PA, USA). TGA of the biomass residues was performed using a TG analyzer (TGA-Q500 series, TA instruments) at standard pressure. About 15 mg of each sample was placed in platinum crucibles and heated linearly at six different heating rates, 5, 10, 15, 20, 25, and 30 °C/min from ambient room temperature to 600 °C. Nitrogen gas at a flow rate of 60 mL/min was used to purge the system and also provided the inert atmosphere for the experiments. All the experimental runs were repeated thrice, and the maximum deviation obtained for the reproducibility was about ±2%, and thus the mean of these values was presented in all the figures used.

### 2.3 Kinetic Modeling

All kinetic principles, models, derivations, and equations utilized are presented in Table 3.

**Table 3:** Equations and Sequence for the Kinetic Analysis of the Four Biomass Materials^a^

1. Kinetic Principles	equation	
reaction rate equation	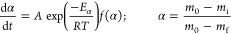	1
	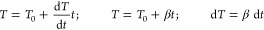	2
	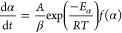	3
	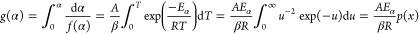	4
2. Isoconversional Models		
Friedman model^15^	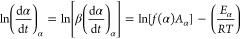	5
Starink model^19^	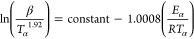	6
Vyazkovin model^20,40^	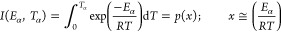	7
	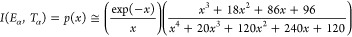	8
	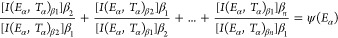	9
3. Reaction Model Derivation		
master plots method^21^	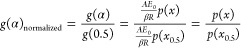	10
4. Pre-exponential Factor Determination		
rearranging the reaction rate equation	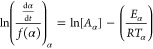	11

a dα/d*t* = rate of reaction conversion (-); *A* = pre-exponential factor (1/s); *R* = ideal gas constant, 8.314 J mol K^–1^; *T* = temperature (K); *f*(α) or *g*(α) = reaction mechanism; *m*_0_, *m*_i_, and *m*_f_ = initial, instantaneous, and final normalized mass in %; β = heating rate (°C/min); *p*(*x*) = temperature integral solution.

## 3 Results and Discussion

### 3.1 Characterization of Biomass

Table 4 provides a summary of the proximate, ultimate, compositional, and heating value analyses for all the four samples tested.

**Table 4:** Biomass Characterization and Compositional Analysis

		soy hull	oat hull	willow	spruce
proximate analysis (wt %)	moisture content	6.5	6.9	7.1	6.7
	volatile matter	78.2	71.5	81.6	83.7
	ash	4.6	5.8	1.4	0.5
	fixed carbon	10.7	15.8	9.9	9.1
ultimate analysis (wt %)	carbon	41.5	43.3	46.2	47.8
	hydrogen	5.7	5.6	5.8	5.9
	nitrogen	1.6	0.6	0.1	0.04
	sulfur	0.1	0.1	0.02	
	oxygen	51.0	50.4	47.8	46.26
component analysis (%)	cellulose	41.0	29.2	55.0	46.3
	hemicellulose	16.2	30.1	13.6	15.5
	lignin	3.5	8.45	14.3	23.6
heating value HHV (MJ/kg)		16.2	17.4	15.4	19.3

It can be observed that all four biomass materials have very high volatile matter ranging from 71.5 to 83.7 wt %, with both agricultural materials yielding slightly less volatile matter content than the two woody biomass materials. Coal generally yields volatile matter up to a maximum of about 45 wt % depending on its type.^41^ The high value of the volatile matter yields for these four materials makes them suitable for various thermochemical processes as the combustibility generally decreases with decreasing volatile matter.^42^ The higher the hemicellulose and cellulose content, the more volatile matter the material has. Therefore, higher volatile matter was observed for the willow and spruce materials. The ash content constitutes the inorganic constituents of the biomass after complete oxidation. The results show higher ash content for soy and oat hulls at 4.6 and 5.8 wt %, respectively, than for willow sawdust and spruce wood at 1.4 and 0.5 wt, % respectively. The difference in ash content may be explained by a higher mineral constituent from accumulating nutrients for the crop growth for the agricultural biomass compared to the woody biomass materials. The fixed carbon content signifies the net char yield for the four samples. Variation in the char yield after pyrolysis is such that soy hull and oat hull yield more than the woody samples. The solid char residues thus follow a similar trend as the ash content for the two biomass classes. The moisture contents for the four biomass samples are within the same range, with the highest being 7.1 wt % for willow sawdust and the lowest for soy hull at 6.5 wt %.

The ultimate analysis shows that the carbon contents for both soy and oat hulls are slightly less than those of the other two biomass materials, while the hydrogen contents for each of the four samples are quite similar. All four samples have nitrogen (0.04–1.6 wt %) and sulfur (0–0.1 wt %) contents which are very low compared to coal in which the sulfur content can be as high as 11 wt %.^41^ The low N and S content implies less corrosion in the gasifier and minimal toxic gas emissions during the thermal degradation of these feedstocks. The heating value analysis showed the highest value for spruce, the feedstock with the lowest ash and highest carbon content. The sum of the wt % of hemicellulose and cellulose is somewhat similar across board; however, the woody biomass generally has a higher cellulose content compared to the nonwoody biomass, with the oat hull having a very low quantity. Conversely, the hemicellulose contents are similar, with the exception of oat hull having a comparatively higher quantity which may be the result of factors that affect biomass composition such as biomass storage, harvesting technique, and geographical location.^43^ Lignin is present in plant cell walls which gives rigidity and imperviousness in terms of binding of the plant cells.^44^ As the woody biomass is generally more rigid, it was observed that the lignin content for both willow (14.3 wt %) and spruce wood (23.6 wt %) would be relatively higher than that for soy (3.47 wt %) and oat (8.46 wt %) hulls. Due to its complex structure, lignin is considered to be a limiting factor in the thermochemical conversion of biomass;^44^ this implies that the lower lignin content of the agricultural biomass material might make it more favorable for thermal degradation than the woody biomass.

### 3.2 Thermal Analysis

Thermal analysis of the four biomass materials is discussed in terms of the effects of heating rate, thermal lag, and the decomposition characteristics using the results derived from the respective TG and differential thermogravimetric (DTG) curves.

#### 3.2.1 Heating Rate and Thermal Lag

The effect of the heating rate on the decomposition (volatiles) and residue (char) yields of the respective biomass materials during the actual TGA are presented in Table 5.

**Table 5:** Experimental Yields of the Four Biomass Samples at Different Heating Rates during TGA

	yield (wt %)							
	soy hull		oat hull		spruce		willow	
heating rate (°C/min)	volatiles	char	volatiles	char	volatiles	char	volatiles	char
5	71.3 ± 0.3	28.7 ± 0.2	74.5 ± 0.3	25.5 ± 0.2	90.0 ± 0.1	9.0 ± 0.1	79.0 ± 0.3	21.0 ± 0.2
10	71.2 ± 0.2	28.8 ± 0.3	75.0 ± 0.2	25.0 ± 0.1	91.6 ± 0.2	8.4 ± 0.2	82.4 ± 0.1	17.6 ± 0.1
15	71.8 ± 0.3	28.2 ± 0.2	74.2 ± 0.3	25.8 ± 0.2	92.8 ± 0.2	7.2 ± 0.2	79.5 ± 0.3	20.5 ± 0.3
20	70.9 ± 0.3	29.1 ± 0.3	76.9 ± 0.4	23.1 ± 0.2	93.2 ± 0.3	6.8 ± 0.2	80.0 ± 0.2	20.0 ± 0.1
25	70.8 ± 0.4	29.2 ± 0.3	75.4 ± 0.2	24.6 ± 0.2	91.4 ± 0.1	8.6 ± 0.2	82.1 ± 0.1	17.9 ± 0.1
30	72.4 ± 1.6	27.6 ± 1.3	75.5 ± 0.2	24.5 ± 0.1	90.5 ± 0.1	9.5 ± 0.2	81.1 ± 0.1	18.9 ± 0.1

The results show higher char yields for soy and oat hulls which are both agriculture-based biomass materials, while more volatile yields were observed for both spruce and willow biomass materials.

**Figure 1:**
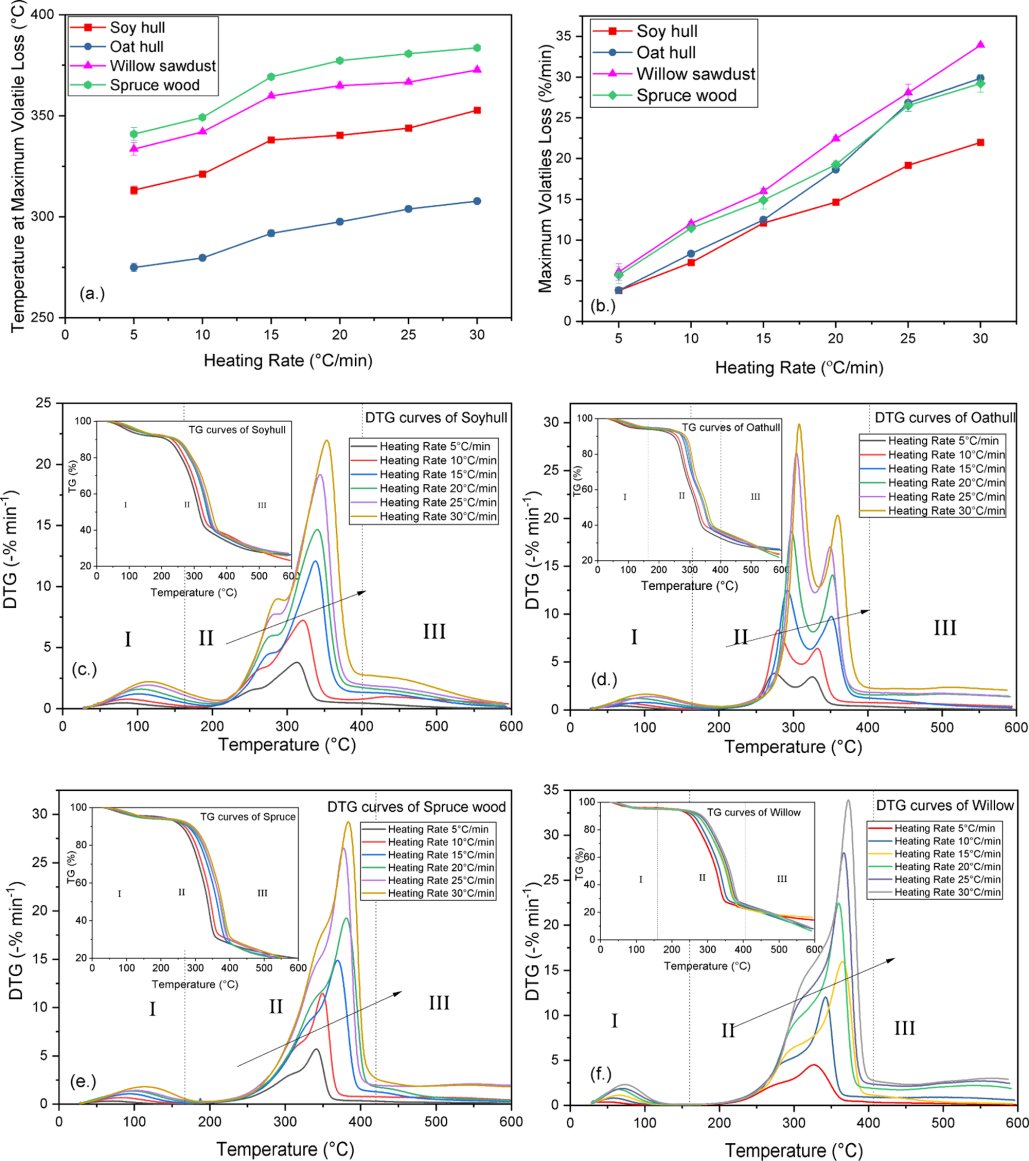
(a) Peak temperature attained for the biomass studied; (b) maximum volatile loss for the biomass studied; (c) DTG and TG profiles of soy hull; (d) DTG and TG profiles of oat hull; (e) DTG and TG profiles of spruce wood; (f) DTG and TG profiles of willow sawdust, all at heating rates 5–30 °C.

Thermal lag was investigated for all four biomass samples *via* a comparison of the heating rate and the maximum temperature observed from the respective DTG curves. Heating rates of 5–30 °C/min were used for all TG experiments, and Figure 1a shows a comparison of the four biomass materials using a plot of the maximum temperature versus the heating rate. It can be observed that the maximum temperature required for complete degradation was higher for the woody biomass as compared to the agricultural biomass which can be easily attributed to the difference in the individual components, as shown in the characterization and compositional analysis in Table 4. Also, as the heating rate increased, there was an increase in the maximum temperature attained for all four biomass samples. This increase in maximum temperature can be ascribed to the effects of thermal lag such that, at a given temperature, a longer duration is required for the biomass to reach a similar mass loss, thus delaying thermal degradation toward higher temperatures. Thermal conductivities of the different biomass materials could also be considered as an additional factor that contributed to the thermal lag observed during thermal degradation, and thus, the biomass sample with the highest thermal conductivity would most likely experience the least thermal lag effect or occurrence.

The start and the end of volatile removal (stages II and III in Figure 1c–f) were also delayed as the heating rate increased; however, the volatiles released increased with an increase in the heating rate, as seen in Figure 1b. This can be attributed to the high heat flux occurring in the higher heating rate which reduces the cohesion of the material and breaks down the structure, thereby speeding up the reactions and resulting in more volatile release. This effect was also observed for other pyrolytic conversion processes involving biomass.^8^

#### 3.2.2 Thermal Decomposition Characteristics

The TG and DTG curves for the four biomass materials are presented in Figure 1c–f. Thermal degradation of each of the four biomass materials can be subdivided into three phases, as numbered in Figure 1c–f, namely, moisture loss, devolatilization and final char formation/removal of secondary gases. The loss of moisture for all four biomass samples takes place between the room temperature and 160 °C. This is represented as phase I in all the DTG curves in Figure 1c–f and by the peaks in this temperature range. Devolatilization occurs in phase II when the biomass is totally devoid of any moisture already removed in phase I. This second phase takes place at a temperature range between 180 and 420 °C. This phase is the main active pyrolysis region where the major bulk of the sample masses is lost. This phase is characterized by two main subsections. The first subsection is generally characterized by a shoulder which represents the decomposition of hemicellulose. The decomposition of hemicellulose as the first major shoulder in the DTG curve has also been reported.^8,45^ This shoulder occurs at a temperature range between 220 and 310 °C for soy hull, spruce wood, and willow sawdust, as can be seen from Figure 1c,e,f. A notable observation is in Figure 1d for oat hulls which unlike the other DTGs has a peak in the first subsection (250–300 °C) rather than a shoulder.

The peak at this first subsection for oat hulls can be explained from the component analysis conducted, as shown in Table 4, where the oat hull sample used contains a higher proportion of hemicellulose than cellulose compared to the other biomass samples. As hemicellulose is the first component, it can indicate that the ignition temperature would be higher for oat hull due to its high composition compared to the other biomass materials and also possibly yield more quantities of oxides of carbon.^46,47^ The second subsection of phase II corresponds to cellulose decomposition, which occurs in the temperature range of 300–410 °C for all four biomass samples. This second subsection represents the maximum peaks for soy hull and the two woody biomass materials. Phase III is the tailing section of the DTG curve which is characterized by lignin decomposition and further degradation of the char residues. However, lignin decomposition occurs slowly over a broader and wider temperature range, including the devolatilization phase from about 200 °C to the end of the DTG curve, although its decomposition is not considered rate-limiting. Some similarities can be observed from the DTG profiles of all four biomass materials. There exists a rightward shift in the curves as the heating rates increased, signified by the arrows on the curves. The maximum heights of the curves also increased as the heating rates increased due to heat transfer, consequently causing thermal lag and some mass-transfer effects.^8,28^ A higher mass loss occurred for both willow and spruce compared to the other two materials due to the higher volatile matter content; higher hemicellulose decomposition occurred for oat hull, as noticed in the first peak shoulder in Figure 1d, due to its higher content compared to all other materials.

### 3.3 Kinetic Analysis

For the kinetic analysis, nonisothermal reaction rate equations were derived from a combination of the Arrhenius law and the law of mass action, as seen in eqs 1–3 of Table 3. Thus, the kinetic analyses of all four biomass samples were carried out to determine the values of the kinetic triplets from two models: a single-step single kinetics model and a multistep kinetics model. These analyses involved solving the kinetic equations in Table 3 by using the data from TG graphs (Figure 1c–f) at six different heating rates. The temperature range below 150 °C was excluded from the kinetic analysis because its contribution to kinetic parameters is negligible.

#### 3.3.1 Apparent Activation Energies

**Figure 2:**
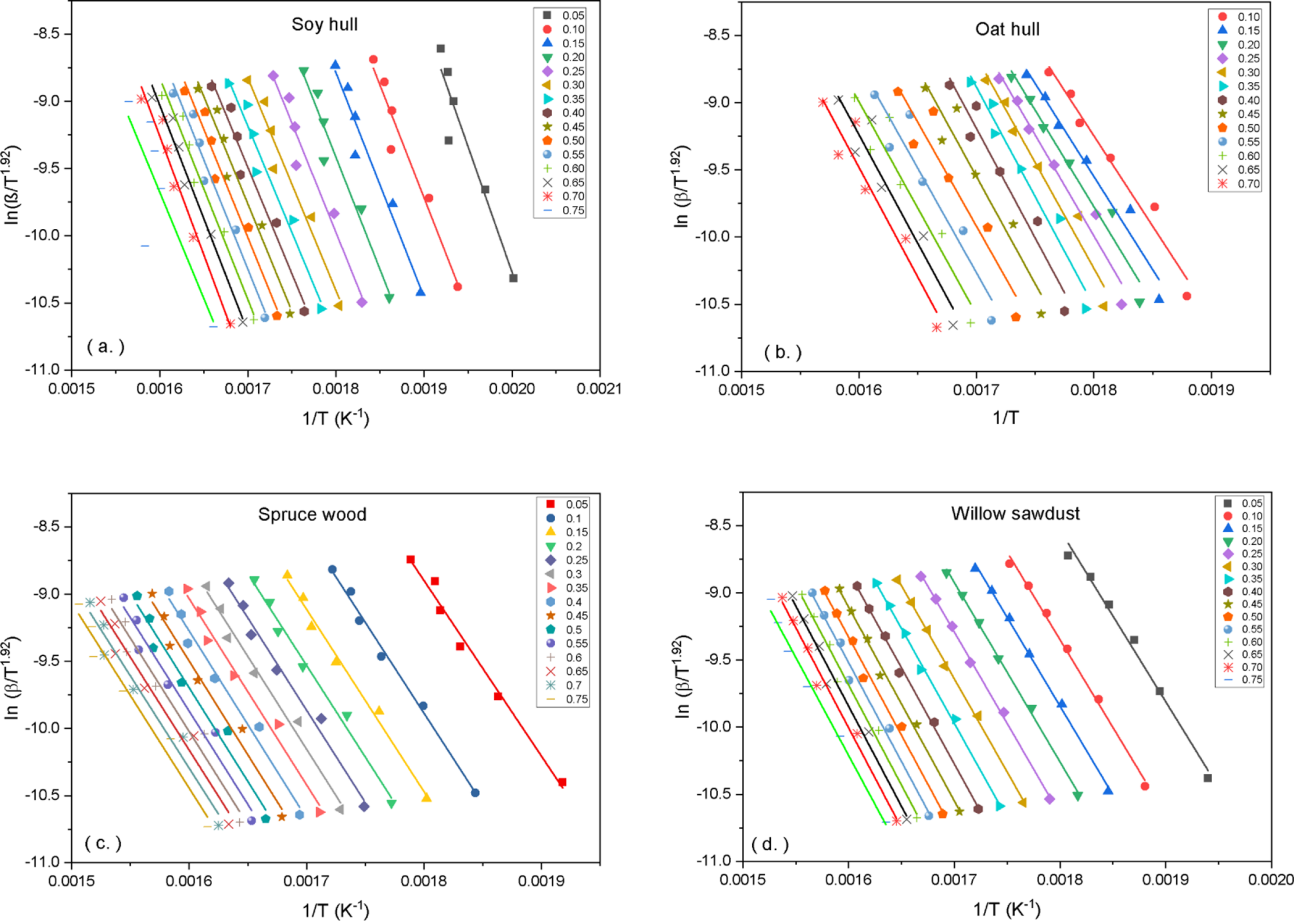
Isoconversional linear regression plots from the Starink model for (a) soy hull, (b) oat hull, (c) spruce, and (d) willow sawdust.

The apparent activation energies (*E*_α_) for the biomass materials were derived using model-free methods by solving the slopes from the linear plots of eqs 5 and 6. The linear regression plots of Starink models for all the four biomass materials at respective conversions are represented in Figure 2a–d. The activation energies derived by the nonlinear Vyazkovin model involves the minimization of the objective function (eq 9)^48^ using the Friedman activation energy values rather than linear regression analysis. This has the added advantage of increased accuracy as it uses numerical integration, as seen from eqs 4, 7, and 8.

#### 3.3.2 Single-Step Single-Kinetic Model

The single-step single-kinetic model assumes that the biomass materials undergo a single global reaction scheme such that a single kinetic model and activation energy describes the thermal decomposition of the materials. Thus, experimental data used in this model conform with the most stable section of the data, with the least deviation from a mean position. Data with poor fits, which usually occur at the beginning and ending of the experiment, are attributed to fluctuations and secondary reactions like ash formation, respectively. Thus, by solving eqs 5, 6, and 9, the activation energies of the biomass can be derived for multiple heating rates from isoconversional kinetics.

The pyrolysis conversion regime for the single-step single-kinetic model has been selected from 0.15 to 0.75 for soy hull, 0.1 to 0.7 for oat hull, and 0.05 to 0.75 for both spruce wood and willow sawdust. The selection was due to the differences in the biomass characteristics, and thus the *R*^2^ values for the linear fit were the highest within the chosen conversion ranges. The *R*^2^ values outside the chosen range were way below 0.8 and so were neglected to fit the single-step single-kinetic model being used. This lack of fit of linear regression plots at 0.8 ≤ α ≤ 0.10 has also been reported by multiple literatures^9,24^ and has been attributed to high heterogeneity from the secondary reactions of char and ash residues.^49^Table 6 shows the slopes, intercepts, and regression values for the two regression models used for only two of the biomass materials used for the sake of brevity. The values of the slopes in Table 6 are presented in thousandth of their actual values as the activation energy would be presented in kJ/mol.

**Table 6:** Isoconversional Regression Plots for Soy Hull and Spruce Wood Using the Friedman and Starink Models

	soy hull						spruce wood					
	Friedman			Starink			Friedman			Starink		
α	intercept	slope	*R*^2^	intercept	slope	*R*^2^	intercept	slope	*R*^2^	intercept	slope	*R*^2^
0.05	27.0	–14.7	0.76	27.2	–18.7	0.89	23.5	–13.7	0.88	14.6	–13.1	0.98
0.10	28.7	–15.9	0.84	22.1	–16.8	0.84	23.7	–14.0	0.87	14.4	–13.5	0.99
0.15	29.7	–16.7	0.96	21.3	–16.7	0.95	23.4	–14.0	0.87	14.0	–13.6	0.99
0.20	28.0	–16.0	0.95	20.7	–16.7	0.94	23.6	–14.2	0.87	13.7	–13.6	0.98
0.25	25.9	–15.1	0.95	19.3	–16.3	0.94	23.8	–14.4	0.87	13.5	–13.7	0.98
0.30	25.7	–15.1	0.95	18.1	–15.8	0.95	23.7	–14.4	0.88	13.3	–13.8	0.98
0.35	26.5	–15.6	0.96	17.7	–15.8	0.95	23.5	–14.5	0.88	13.1	–13.9	0.99
0.40	27.1	–16.0	0.96	17.4	–15.8	0.95	23.5	–14.5	0.88	13.0	–14.0	0.98
0.45	27.5	–16.4	0.96	17.4	–16.0	0.95	23.3	–14.5	0.86	12.8	–14.0	0.98
0.50	27.8	–16.7	0.97	17.4	–16.1	0.95	23.0	–14.4	0.83	12.6	–13.9	0.97
0.55	28.2	–17.0	0.97	17.4	–16.3	0.96	22.6	–14.1	0.81	12.3	–13.9	0.97
0.60	29.1	–17.7	0.97	17.6	–16.5	0.96	22.2	–13.9	0.81	12.2	–13.9	0.96
0.65	31.0	–19.0	0.99	17.9	–16.9	0.97	21.8	–13.7	0.82	11.9	–13.8	0.98
0.70	35.4	–21.9	0.92	19.0	–17.6	0.96	21.8	–13.8	0.87	11.6	–13.7	0.97
0.75	27.7	–17.7	0.10	15.6	–15.8	0.90	22.5	–14.4	0.94	11.6	–13.8	0.96
0.80	0.5	–1.3	0.25	–6.3	–2.2	0.22	23.1	–15.4	0.31	12.3	–14.4	0.99
0.85	4.5	–4.4	0.11	–8.9	–0.6	0.25	–1.2	–0.2	0.25	0.3	–6.8	0.33
0.90	5.7	–5.6	0.06	–6.8	–2.2	0.22	3.7	–4.3	0.05	–4.7	–3.7	0.13
0.95	8.2	–8.1	0.02	–5.8	–3.2	0.20	7.8	–7.9	0.08	–6.0	–3.1	0.14

Seeing the haphazard nature of the lower (α < 0.15) and higher end (α > 0.70) conversion values of the kinetic parameters, the isoconversional analysis for the single-step single-kinetic model restricted the conversion range to between 0.15 and 0.75. This range of minimum deviation of *E*α values is also recommended by the studies in refs (24) and (50)

**Figure 3:**
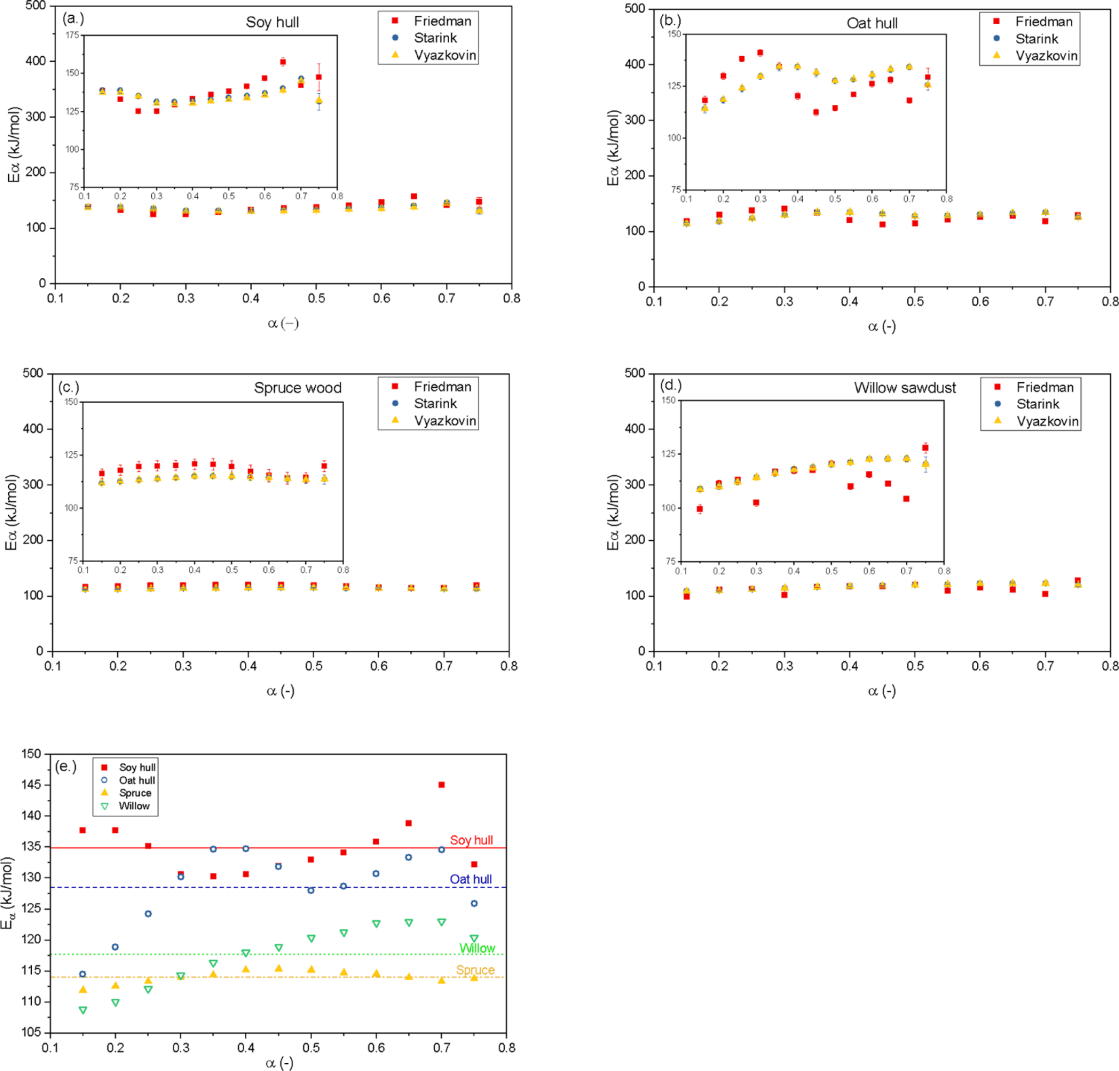
Apparent activation energies at different conversions for (a) soy hull, (b) oat hull, (c) spruce, (d) willow, and (e) mean *E*_α_ combined.

The apparent activation energies from three selected isoconversional models are plotted versus conversion and presented in Figure 3a–d. It can be observed that for all the three models employed, there is negligible variation in the mean values of activation energies. The Friedman model had higher mean *E*_α_ values within the conversion range considered for both soy hull and willow while having less values for both oat and spruce compared to the other two models. The differential method utilized by the Friedman model makes it susceptible to some errors during data computation and as such could render computed values sparsely distributed around the mean. Thus, the standard error values calculated for the Friedman model were a little higher compared to the other two models. The results from the Vyazkovin model produced the least magnitude in errors compared to the other models as it is based on the minimization of the objective function in eq 9, with the initial parameter set to the values obtained from the Friedman model. The objective function uses the fourth-order Senum–Yang expression (eq 8)^40^ for the temperature integral which results in less errors compared to other models. Therefore, the values obtained from the Vyazkovin model were selected and used in deriving the final models. The Vyazkovin model results for apparent activation energies and conversion range selected for all the four biomass materials are plotted in Figure 3e, with the horizontal lines depicting the mean *E*_a_ values. By comparing the individual materials, it was found that the agricultural biomass materials soy hull (134.9 kJ/mol) and oat hull (128.5 kJ/mol) had higher values for their activation energies than the woody biomass materials, spruce (114.0 kJ/mol) and willow (117.7 kJ/mol) selected. The higher activation energies could be due to the way the components of the materials are structured and the manner the various bonds are arranged. The results of proximate analyses (Table 4) also show higher ash contents for the agricultural biomass which may contain minerals that elevate the energy barrier, thus increasing the activation energy. Several literatures have presented *E*_α_ values for isoconversional models for various biomass materials from agriculture to woody and some from other sources. The results from TG analysis carried out by Huang et al^29^ on soybean straw gave *E*_α_ values between 154.2 and 156.2 kJ/mol. Prins et al^51^ performed weight loss kinetics on torrefied willow wood, with the *E*_α_ results lying between 114.2 and 151.7 kJ/mol. The *E*_α_ value of 189 kJ/mol was derived by Miranda et al^52^ from the thermal decomposition of soybean hull cellulose.

##### 3.3.2.1 Single-Step Reaction Models

**Figure 4:**
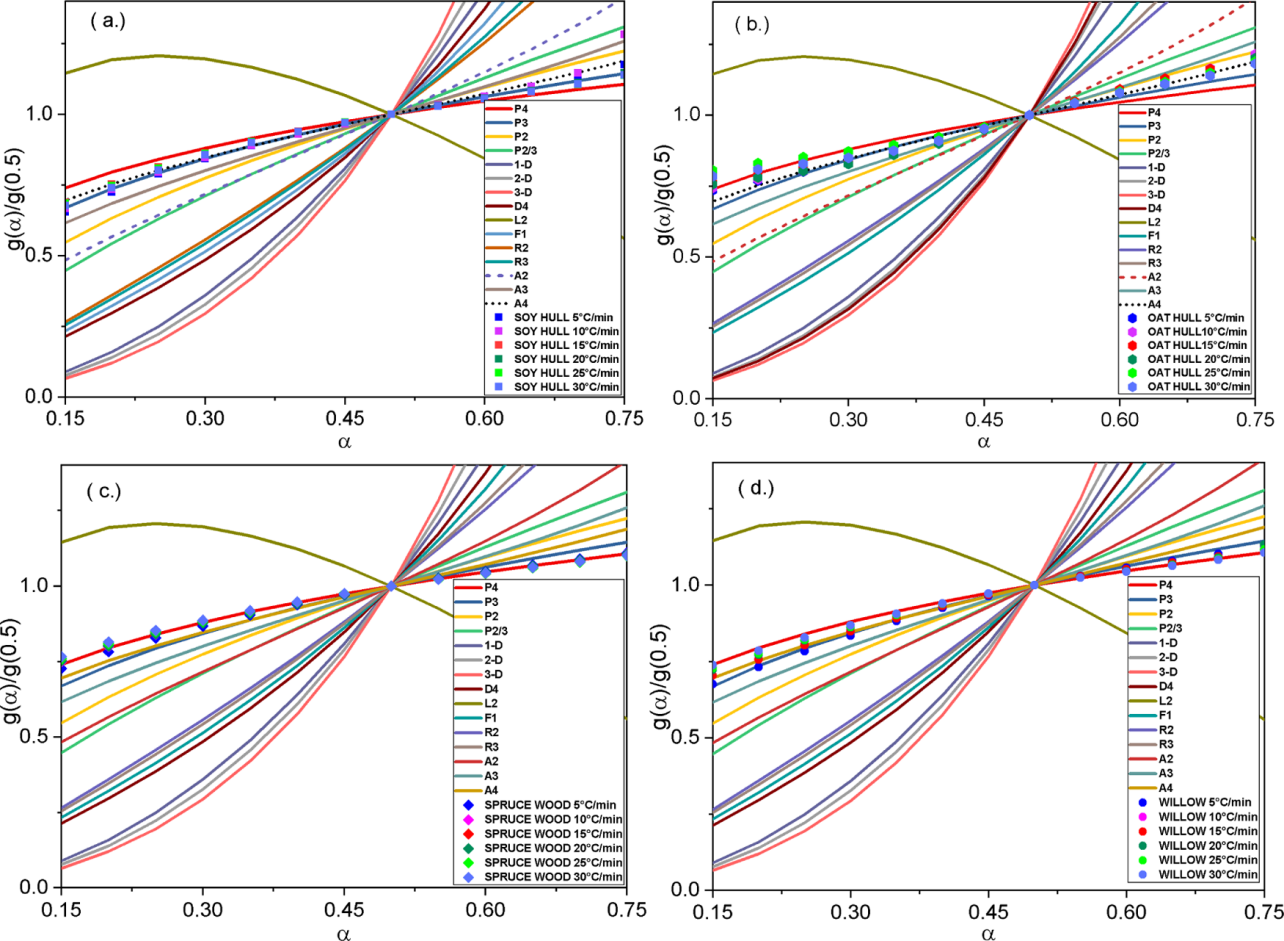
Integral master plot for different theoretical models using single-step models for (a) soy hull, (b) oat hull, (c) spruce wood, and (d) willow sawdust.

The reaction model for single-step single-kinetics was derived for each of the four biomass materials using the generalized integral master plots *via*eq 10. The experimental *g*(α) are derived by substituting the values of the known constants β, *R*, and the other kinetic parameters, *A*, *E*_0_ (mean value of the apparent activation energy based off the Vyazkovin model), and *p*(*x*). The plots of the experimental *g*(α) values were then compared with the theoretical plots. The integral master plots are illustrated in Figure 4a–d for the respective biomass materials.

For all the biomass materials considered, it can be seen that at the conversion range (α) between 0.15 and 0.75, the experimental data mostly correspond with either the power (P*n*) or the Avrami–Erofeev (A*n*) mechanisms based on the shapes of the integral master plots. Both the power law (P*n*)  and Avrami–Erofeev (A*n*) [*f*(α) = *n*(1 – α)[−ln(1 – α)]^*m*^*n* = 1/1 – *m*] models follow a nucleation or nuclei growth mechanism. Other literatures^9,27^ have explained similar decomposition phenomena occurring *via* the nuclei growth mechanism. Thus, the thermal decomposition of a solid to produce a new product and a gas can follow a nucleation mechanism as a new product phase is being formed at the nucleation sites of the initial reactant. The new nucleation sites grow, and the growth rate could follow an order which depends on the type and nature of the solid reactants.^53^ The data for the biomass decomposition were fitted to these models using the Levenberg–Marquardt algorithm at five different heating rates and then subsequently optimized by maximizing the *R*^2^ value of the biomass decomposition data.

**Figure 5:**
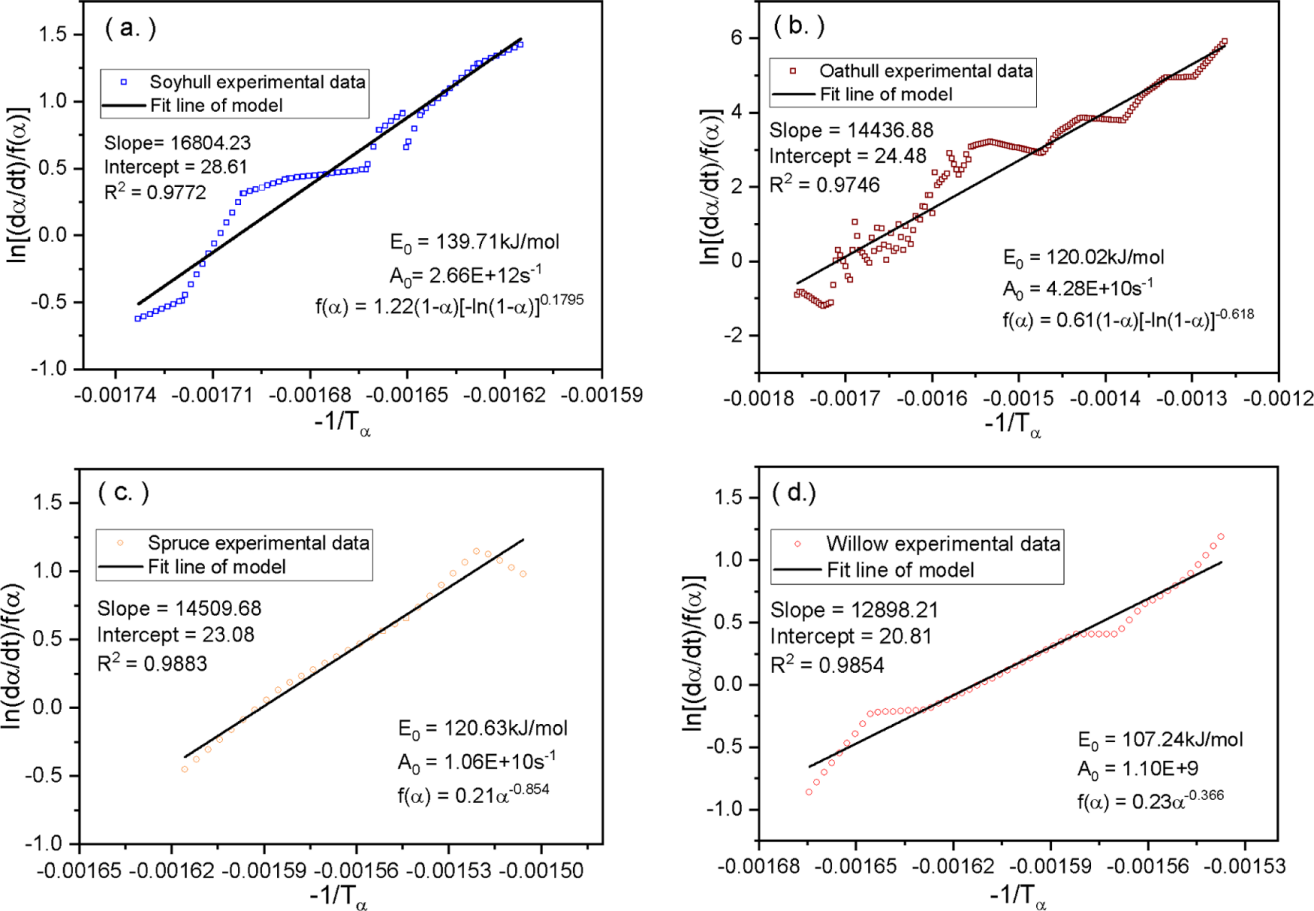
Single-step kinetic fitting results for (a) soy hull, (b) oat hull, (c) spruce, and (d) willow.

Figure 5 shows the results from the fitting and optimization of the corresponding power and Avrami–Erofeev models to the decomposition kinetics of the four biomass materials using the single-step single-kinetic model. It can be observed that both agricultural biomass materials, the soy and oat hull samples, have data that best fit the Avrami–Erofeev models, while the spruce and willow biomass materials follow the power mechanism within the conversion ratios considered. Each figure gives the value of the activation energy and intercept for the respective biomass. The activation energies for each of the four materials from the models are 139.7 kJ/mol for soy hull, 120.0 kJ/mol for oat hull, 120.6 kJ/mol for spruce wood, and 107.2 kJ/mol for willow sawdust. It can be observed that the values of activation energies derived from the model fitting are quite comparable with the isoconversional values derived earlier in Sections 3.3.1 and 3.3.2, with the least deviation occurring for soy hull at +3.6% and a maximum of −8.85% for sawdust. According to ref (24), the recommended maximum deviation value for the activation energies should be within a tolerance level of ±30%; thus, the values derived here are well within the acceptable range. Substituting the intercepts of the regression curves (Figure 5) and *f*(α) models of the respective biomass in eq 5 facilitated the calculation of frequency factors. The values of the frequency factors were calculated to be 2.66 × 10^12^ s^–1^ for soy hull, 4.28 × 10^10^ s^–1^ for oat hull, 1.06 × 10^10^ s^–1^ for spruce wood, and 1.1 × 10^9^ s^–1^ for willow sawdust. The *R*^2^ correlation values of linear regression for the model used are quite high (generally above 0.97), with the value as high as 0.99 for spruce wood. This shows that the model used suitably follows the decomposition mechanism.

##### 3.3.2.2 Model Validation

**Figure 6:**
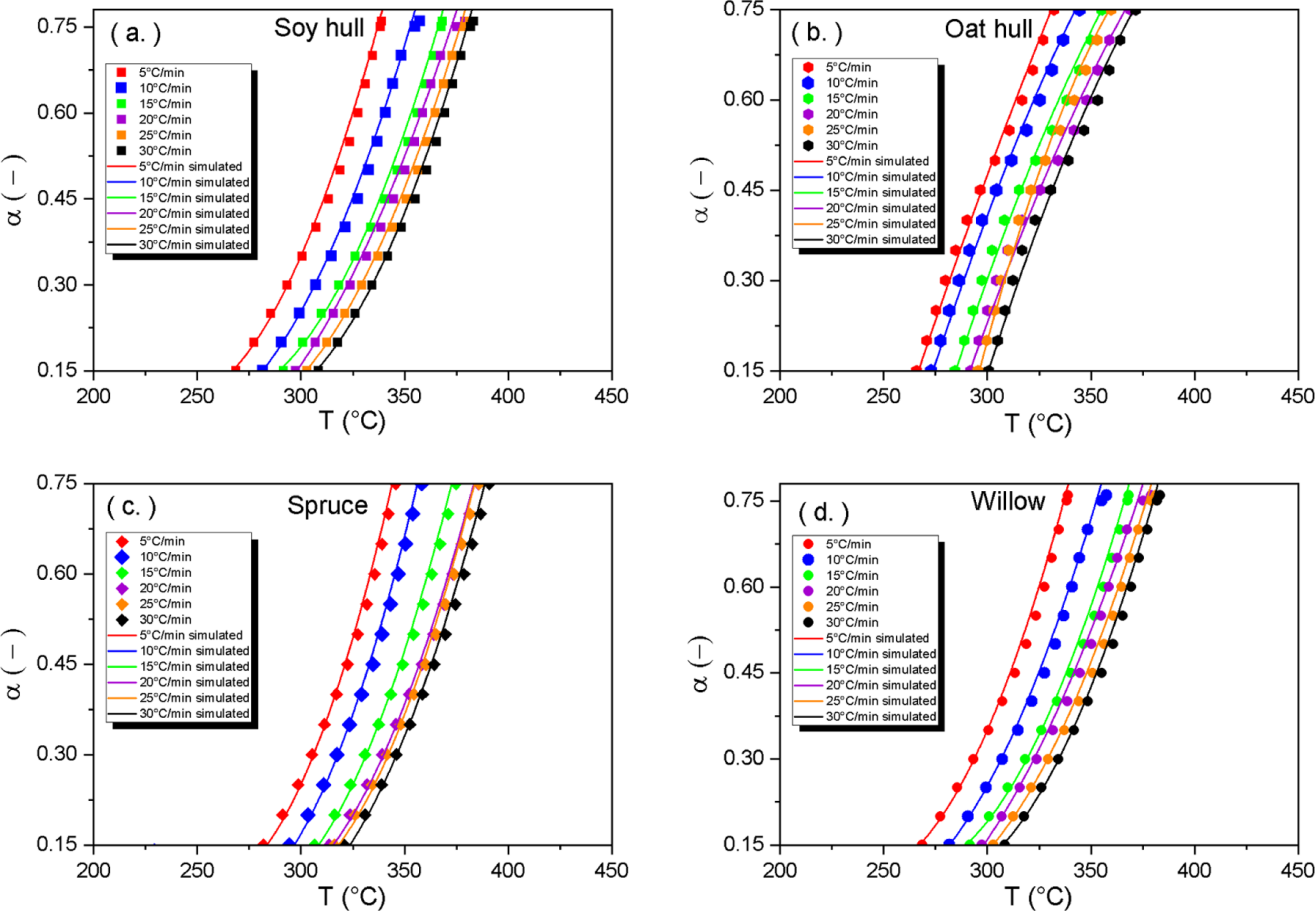
Experimental *vs* simulated data plots of single-step reaction mechanism for (a) soy hull, (b) oat hull, (c) spruce, and (d) willow.

To validate the results derived from the single-step models describing the four biomass materials at different heating rates, the fourth-order Runge–Kutta method was used from the MATLAB ODE45 solver.^54^ The comparison between the experimental and simulated data is shown in Figure 6.

The simulated curves fit closely with the experimental data within the conversion ranges (0.15–0.75) chosen for each of the biomass materials. Because the simulated and experimental data fit poorly at lower conversion ranges below 0.15 and above 0.75, a multistep approach is therefore considered to determine the kinetic parameters in the regions within these ranges.

#### 3.3.3 Multistep Reaction and Kinetics

**Figure 7:**
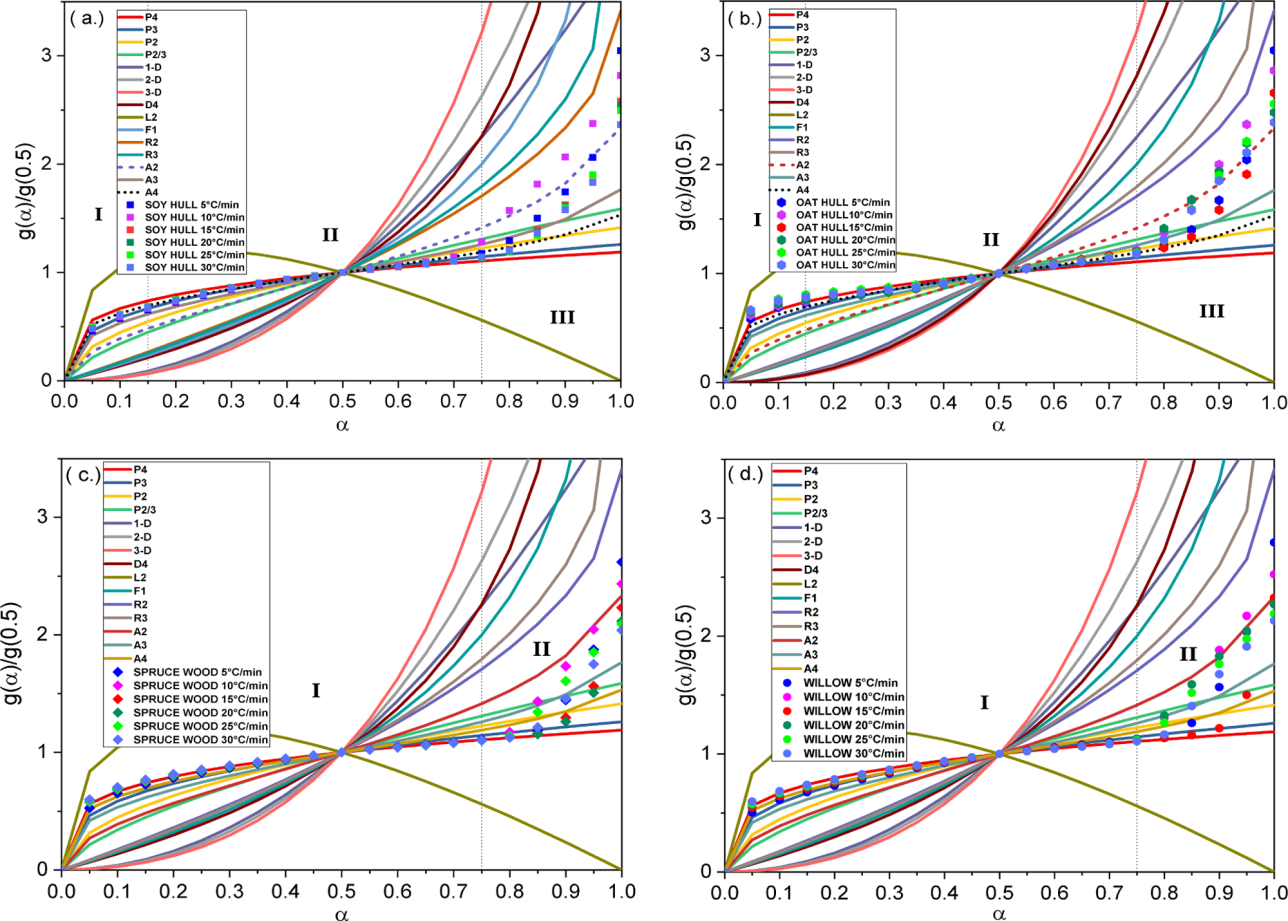
Integral master plot for different theoretical models using multiple step models for (a) soy hull, (b) oat hull, (c) spruce wood, and (d) willow sawdust.

The multistep reaction and kinetic model used in this work suggests that the degradation of the biomass occurs in a stepwise consecutive breakdown of each of the major biomass pseudocomponents, each step having its own unique kinetic expression based on the particular component decomposed. Unlike the single-step kinetics, which assumes that secondary reactions constitute the early and later stages of the whole reaction, the multistep model considers both the initial part (0 ≤ α ≤ 0.15) and the later part (0.75 ≤ α ≤ 1). The shape of the master plots in Figure 7 from the multistep kinetic model suggests the number of stages/steps involved to reach the four different biomass materials. Thus, each major step is assigned its own kinetic expression.

As seen in Figure 7a,b, the integral master plots can be demarcated into three distinct sections: α < 0.15, 0.15 < α < 0.75, and α > 0.75. Each of these sections can be assumed to have its own unique reaction model *f*(α) based on the shape of the integral plot. The different range in isoconversional *r*-values for each of the three sections demarcated gives further credence to this multistep mechanism assumption. The intercept, *r*-values, mean of the slope, and subsequently the apparent activation energies are consistent within each unique demarcation. This variation in kinetic parameters for each section substantiates the assumption of a multiple step kinetics and helps to detect the reaction mechanism and pre-exponential factors. The multistep integral master plot for soy hull residues is shown in Figure 7a. At conversion values less than 0.15, the experimental plots for the soy residues at different heating rates coincide with the theoretical P3 power model. Conversion values between 0.15 and 0.75 correspond and coincide with the theoretical A4 Avrami–Erofeev model. The power and Avrami–Erofeev models are both nucleation models where the nuclei growth describes the biomass decomposition process. The power model assumes that the nuclei growth is constant, while the Avrami–Erofeev model assumes that there exist some limitations to the nuclei growth.^53^ The Avrami–Erofeev A2 and contracting volume R2 models describe the later end of the plots at a conversion range above 0.75. The A2 model is a lower order magnitude nucleation model, while R2 is a geometrical area contraction model where the degradation rate is governed by the reaction interface progression from the surface to the center of the material.^53^

The master plots for the oat hull bioresidues are presented in Figure 7b. The model plots for the oat hull residue have a similar trend as the soy hull residues. However, unlike soy hull which coincides with the P3 power model with conversion ratios below 0.15, the experimental curves for the oat hull residues follow the P4 power law trend within this conversion range. The slight difference in the α < 0.15 range may be attributed to the higher hemicellulose content of the oat hull bioresidue compared to the soy hull (see Table 4) and hence the slightly higher order of the power model present for the oat hull.

Figure 7c,d shows the master plots for the wood-based biomass residues. Both plots show demarcation into two distinct segments unlike the three segments observed for the agricultural bioresidues. Figure 7c shows the plots for spruce wood with two distinguishable sections, one within the conversion range of α ≤ 0.75 and the other at the conversion range of α > 0.75. The first section of the experimental plot for the spruce wood bioresidue coincides with the theoretical P4 power model from the initiation of decomposition until about 75% of the spruce components are decomposed. Beyond the 0.75 conversion of the spruce biomass, the mechanism of decomposition followed the Avrami–Erofeev A2 model. The willow bioresidue followed a similar pattern as the spruce wood, as seen in Figure 7d. Willow sawdust coincides with the P4 power model at conversion ranges below 0.75. The mechanism of decomposition for willow in the conversion range above 0.75 coincides with the A2 model in a similar fashion to that for the spruce wood materials. The distinction into two separate segments is consistent for both wood-based biomass residues. The woody biomass residues tend to taper toward the contracting cylinder and diffusion models at the very tail end of the conversion but maintain the nucleation model for the most part of the whole pyrolytic conversion process.

The observation that different sections of the experimental master plots conform with different theoretical models, as seen in the various plots in Figure 7, suggests that the mechanism of decomposition for each of the four bioresidues is a complex process involving multiple components and multiple stages. Findings from previous studies^25,30^ corroborate the multistage approach used in this study. These stages should therefore be assigned their individual kinetic parameters for the appropriate evaluation of the mechanism of the multicomponent decomposition.

Section 3.2.2 explains the thermal decomposition characteristics of the four bioresidues studied and the temperature range of decomposition for each individual component. The temperature and conversion range for the component decomposition observed for the four biomass materials used conform with those from literature studies.^55^

By observing the stages and components of the bioresidues, much of the initial decomposition would conveniently fit in with hemicellulose. By modeling the detailed pathways of hemicellulose decomposition, including intermediates and products,^56^ hundreds of reactions of 114 species were carried out, concluding that hemicellulose decomposes *via* a mechanism similar to that of cellulose. Also, several findings^9,57,58^ have proven that cellulose decomposes through the nucleation kinetic model. It can thus be deduced that both hemicellulose and cellulose should decompose *via* the nucleation kinetic model, as evidenced from the results obtained herein. Lignin, as the third major component, shows a more complex pattern as its mechanism is a combined effect of any one or more of nucleation, geometric contraction, and diffusion.^27^ Thus, its decomposition is assumed to span across all conversion ratio ranges,^9,55^ although a little more may be attributed to the latter conversion range, as can be seen in the sharp changes observed in the integral plots (Figure 7) after the α > 0.80 conversions for soy and oat hulls. The woody spruce and willow contain more lignin content compared to the soy and oat hull materials. It can be observed that the lignin content of the wood-based biomass materials is more distributed and thus decomposed within the earlier conversion ratios, with fewer left at the α > 0.80 conversion range. This explains the two distinct stages observed for spruce and willow compared to the three noticed in the agriculture-based materials.

The kinetic parameters for the multistep approach are derived by treating each step as distinct from each other. Each step is assigned its own kinetic triplet based on the reaction mechanisms derived and discussed in Section 3.3.3. By substituting the reaction models observed from Figure 7 in the form of *f*(α) into eqs 1, 3, or 5, the pre-exponential factors can be obtained by rearranging to give eq 11 and solving for the intercept of eq 11 as all other parameters are already known. The results are summarized in Table 7.

**Table 7:** Results of Kinetic Parameters Derived from the Multistep Model for the Four Biomass Residues

soyhull		
kinetic parameters		
stage I (α < 0.15)	stage II (0.15 < α < 0.75)	stage III (α > 0.75)
*E*α = 146.3 kJ/mol	*E*_α_ = 134.1 kJ/mol	*E*_α_ = 39.7 kJ/mol
*A* = 2.89 × 10^12^ s^–1^	*A* = 1.65 × 10^12^ s^–1^	*A* = 2.91 × 10^8^ s^–1^
*f*(α) = P3—power law	*f*(α) = A4—Avrami model	*f*(α) = A2/R2—Avrami/geometric contraction model

oathull		
kinetic parameters		
stage I (α < 0.15)	stage II (0.15 < α < 0.75)	stage III (α > 0.75)
*E*_α_ = 109.5 kJ/mol	*E*_α_ = 128.5 kJ/mol	*E*_α_ = 35.9 kJ/mol
*A* = 4.75 × 10^10^ s^–1^	*A* = 1.41 × 10^12^ s^–1^	*A* = 3.57 × 10^8^ s^–1^
*f*(α) = P4—power law	*f*(α) = A4—Avrami model	*f*(α) = A2/R2—Avrami/geometric contraction model

spruce wood	
kinetic parameters	
stage I (α < 0.75)	stage II (α > 0.75)
*E*_α_ = 113.4 kJ/mol	*E*_α_ = 72.6 kJ/mol
*A* = 1.12 × 10^10^ s^–1^	*A* = 6.02 × 10^9^ s^–1^
*f*(α) = P4—power law	*f*(α) = A2—Avrami model

willow	
kinetic parameters	
stage I (α < 0.75)	stage II (α > 0.75)
*E*_α_ = 115.9 kJ/mol	*E*_α_ = 32.9 kJ/mol
*A* = 3.47 × 10^9^ s^–1^	*A* = 6.25 × 10^5^ s^–1^
*f*(α) = P4—power law	*f*(α) = A2—Avrami model

## 4 Conclusions

The thermal degradation kinetics of biomass residues were investigated. The maximum weight loss occurred at the second region for all residues, and the temperature limit where these losses occurred was higher for the woody biomass. Nucleation mechanisms best described the single-step models posited for the materials. Using the multistep kinetic model, the agricultural biomass degraded in three, while wood-based biomass degraded in two, consecutive stages. Although nucleation was observed across board, they differ in terms of range and the *f*(α) or *g*(α) mechanisms. These results demonstrate that more than one kind of model may be required to interpret the kinetics of biomass materials.
